# Genetic Evidence and Host Immune Response in Persons Reinfected with SARS-CoV-2, Brazil

**DOI:** 10.3201/eid2705.204912

**Published:** 2021-05

**Authors:** Natalia Fintelman-Rodrigues, Aline P.D. da Silva, Monique Cristina dos Santos, Felipe B. Saraiva, Marcelo A. Ferreira, João Gesto, Danielle A.S. Rodrigues, André M. Vale, Isaclaudia G. de Azevedo, Vinícius C. Soares, Hui Jiang, Hongdong Tan, Diogo A. Tschoeke, Carolina Q. Sacramento, Fernando A. Bozza, Carlos M. Morel, Patrícia T. Bozza, Thiago Moreno L. Souza

**Affiliations:** Fundação Oswaldo Cruz (Fiocruz), Rio de Janeiro, Brazil (N. Fintelman-Rodrigues, A.P.D. da Silva, M.C. dos Santos, F.B. Saraiva, M.A. Ferreira, J. Gesto, I.G. de Azevedo, V.C. Soares, C.Q. Sacramento, F.A. Bozza, C.M. Morel, P.T. Bozza, T.M.L. Souza);; Universidade Federal do Rio de Janeiro, Rio de Janeiro (D.A.S. Rodrigues, A.M. Vale, V.C. Soares, D.A. Tschoeke);; MGI Tech Co., Ltd., Shenzhen, China (H. Jiang, H. Tan);; D’Or Institute for Research and Education (F.A. Bozza)

**Keywords:** COVID-19, coronavirus disease, SARS-CoV-2, severe acute respiratory syndrome coronavirus 2, viruses, respiratory infections, zoonoses, reinfection, next-generation sequencing, humoral response, Brazil

## Abstract

The dynamics underlying severe acute respiratory syndrome coronavirus 2 (SARS-CoV-2) reinfection remain poorly understood. We identified a small cluster of patients in Brazil who experienced 2 episodes of coronavirus disease (COVID-19) in March and late May 2020. In the first episode, patients manifested an enhanced innate response compared with healthy persons, but neutralizing humoral immunity was not fully achieved. The second episode was associated with different SARS-CoV-2 strains, higher viral loads, and clinical symptoms. Our finding that persons with mild COVID-19 may have controlled SARS-CoV-2 replication without developing detectable humoral immunity suggests that reinfection is more frequent than supposed, but this hypothesis is not well documented.

Confirmed cases of severe acute respiratory syndrome coronavirus 2 (SARS-CoV-2) have surpassed 110 million, along with 2.5 million deaths by 2019 coronavirus disease (COVID-19) (*1*). New waves of the pandemics in different Northern and Southern Hemisphere countries provide evidence that herd immunity might not have been fully achieved and that new variants could escape the response to natural infection (*2*,*3*).

Although there is evidence of the generation of B and T memory cells to SARS-CoV-2 proteins after infection (*4*,*5*), it has also been documented that neutralizing seroconversion is heterogeneous among the population (*6*). Even for those who seroconvert, the sustainability of the immune response, as judged by IgG level, might decay after the primary exposure to coronaviruses (*7*–*9*). Cases of reinfection by SARS-CoV-2 can be associated with the absence of neutralizing serologic titers, diminishment of immunoglobulin titers after primo-infection, or viral polymorphisms to escape the host SARS-CoV-2 immune response (*10*–*16*).

To better understand the dynamics of the immune and virological responses in mild cases of COVID-19 that might predispose patients to reinfection, we continuously followed up with patients for potential exposure to SARS-CoV-2. For 2 patients, reinfection was documented. The National Review Board of Brazil approved the study protocol (Comissão Nacional de Ética em Pesquisa [CONEP] 30650420.4.1001.0008), and informed consent was obtained from all participants or patients’ representatives.

## Materials and Methods

### Ethics and Study Population

During March–December 2020, the COVID-19 research task force screened a group of 30 participants weekly, independent of any symptoms, for SARS-CoV-2 detection by RT-PCR in nasopharyngeal swab specimens. If any of these participants exhibited positive results, or members of their households experienced signs or symptoms of COVID-19, they were invited to participate in the study and follow-up. At baseline and follow-up, we collected plasma, serum, and nasopharyngeal swab samples biweekly or at longer intervals if the patient was unavailable (Table). Households were included upon their request to be tested for SARS-CoV-2. Among the participants, 4 exhibited >1 episode of mild self-limiting COVID-19 with positive RT-PCR. For comparison, we included age-matched controls from the same group of participants and city in which the patients lived, Rio de Janeiro, Brazil. Controls were composed of 5 persons negative for SARS-CoV-2 throughout the investigated period. 

**Table:** Characteristics of patients reinfected with severe acute respiratory syndrome coronavirus 2, Brazil, 2020*

Characteristic	Patient A	Patient B	Patient C	Patient D
Primo-infection				
Sex	M	F	M	F
Age, y	54	57	34	34
Concurrent conditions	None	Discoid lupus erythematosus	None	None
Date of symptom onset	March 21	March 26	Asymptomatic	March 31
Symptoms	Headache	Mild diarrhea	No	Mild diarrhea
N1 RT-PCR, log_10_ copies/mL	5.12	3.21	3.83	3.01
Date conducted	March 23	March 24	March 24	April 2
RNP RT-PCR (internal control), C_t_	26.5	26.66	27.41	28.48
Serology†	IgM, IgA, IgG detected	IgM, IgA, IgG detected	IgM, IgA, IgG detected	IgM, IgA, IgG detected
PRNT_90_/25 uL†	<1:4	<1:4	<1:4	<1:4
Sequencing	Not enough sample	Emerging clade 19A	Emerging clade 20B	Not enough sample
ID	N/A	EPI_ISL_636834	EPI_ISL_636836	NA
Second infection				
Date of onset illness	May 25	May 26	May 27	May 30
Symptoms	Fever, dry cough, tiredness, body ache, anosmia, ageusia	Fever, diarrhea, headache, body ache, anosmia, ageusia	Fever, nausea, tiredness, headache, body ache	Dry cough, diarrhea, tiredness, headache, body ache, anosmia, ageusia
RT-PCR, log_10_ copies/mL	7.31	7.42	5.18	9.61
Date conducted	May 29	May 29	May 29	May 29
RNP RT-PCR internal control	24.6	27.06	28.12	24.5
Serology results‡	IgM, IgA, IgG detected	IgM, IgA, IgG detected	IgM, IgA, IgG undetectable	IgM, IgA, IgG undetectable
PRNT_90_/25 uL‡	1:16	<1:4	<1:4	<1:4
Sequencing	Emerging clade 20B	Emerging clade 20B	Emerging clade 20B	Emerging clade 20B
Accession ID	EPI_ISL_636737	EPI_ISL_636835	EPI_ISL_636837	EPI_ISL_636838
Follow-up				
Serology§	IgM, IgA, IgG detected	IgM, IgA, IgG detected	IgM, IgA, IgG detected	IgM, IgA, IgG detected
PRNT_90_/25 uL§	1:128	1:32	1:64	1:64
Serology results¶	IgM, IgA, IgG detected	IgM, IgA, IgG detected	IgM, IgA, IgG detected	IgM, IgA, IgG detected
PRNT_90_/25 uL	1:64	1:16	1:8	1:8

*N1, nucleocapside gene; NA, not available; PRNT_90_, 90% plaque-reduction neutralization test; RNP, human RNase P gene; RT-PCR, reverse transcription PCR.  †Tests conducted March 27. ‡Tests conducted June 3.  §Tests conducted July 9. ¶Tests conducted August 10.

### Measurement of Serum SARS-CoV-2 Antibodies and Plasma Cytokine Levels

For quantitative analysis of SARS-CoV-2 spike protein IgM, IgA, and IgG antibodies, we performed the S-UFRJ test developed at Universidade Federal do Rio de Janeiro (R.G.F. Alvim et al., unpub. data, https://doi.org/10.1101/2020.07.13.20152884) (Appendix).

We collected plasma samples in tubes containing EDTA. We used commercial ELISA kits from R&D Systems (https://www.rndsystems.com) to measure cytokines and chemokine (Appendix).

### Molecular Diagnosis

To determine serum titers to block SARS-CoV-2 infection, we performed miniaturized plaque-reduction neutralization test (PRNT) (Appendix). SARS-CoV-2 RNA has been detected in accordance with the US Centers for Disease Control and Prevention (CDC) recommendation (*17*). We used the standard curve method for virus quantification, using synthetic RNA for gene N (Microbiologics, https://www.microbiologics.com). We compared cycle thresholds (C_t_) for the target gene to those obtained with different cell amounts (10^7^–10^2^), for reaction calibration (Appendix).

### Genomic Analysis

We extracted total viral RNA from nasopharyngeal swabs using QIAamp Viral RNA (QIAGEN, https://www.qiagen.com), with minor modifications (*18*) (Appendix). We performed an amplicon-based enrichment strategy using the ATOPlex SARS-CoV-2 Full-Length Genome Panel version 1.0 (MGI Tech Co., https://en.mgi-tech.com; donated by the vendor). Single-stranded circular DNA library pools were converted to DNA nanoballs by rolling circle amplification and submitted to pair-end sequencing (100 nt) on the MGISEQ-2000 platform (recently named DNBSEQ-G400; MGI Tech Co. Ltd.).

We quality-scored, filtered, trimmed, and assembled genomic sequences in contigs through a validated workflow for SARS-CoV-2 (*19*). Genomes were aligned with MAFFT (*20*) or ClustalW (*21*), and phylogenies were constructed with MEGA version 7.0 (*22**,**23*), using the Jukes-Cantor model for maximum-likelihood estimates by applying neighbor-joining and BioNJ algorithms (*24*), or by MrBayes version 3.2.7 (http://nbisweden.github.io/MrBayes) (*25**,**26*) with a relaxed clock model with a priori model testing using the gamma rates and invariant sites nucleotide substitution model, selected by jModelTest version 1.6 http://darwin.uvigo.es/software/jmodeltest.htm. We visualized and edited the tree with FigTree version 1.4.2 (http://tree.bio.ed.ac.uk). We determined SARS-CoV-2 clades using the Nextclade software, beta version 0.14 (https://clades.nextstrain.org). To categorize mutations and polymorphisms, we aligned the SARS-CoV-2 reference genome Wuhan-Hu-1 (GISAID EPI ISL no. 402125; https://www.gisaid.org) to our sequences. The original sequences used in this work are publicly available on https://nextstrain.org/ncov: GISAID EPI ISL nos. 636737, 636834–636838. The dataset included in the analysis contained representative sequences of the emerging clades associated with our sequences, 19A and 20B, as well as sequences from the genome 20A as a negative control (Appendix Table 1).

## Results

Among the households of the COVID-19 research task force, a 54-year-old man (patient A) requested an RT-PCR test for SARS-CoV-2 on March 23 because of a recurrent headache on the prior 2 days. He also had previous contact with a symptomatic co-worker returning from travel who refused to be tested. Patient A had a detectable viral load (C_t_ 27.41) of ≈10^5^ copies/mL in nasopharyngeal swab samples (Table). Although patient B, a 57-year-old woman with a previous history of discoid lupus erythematosus, was in self-isolation, she was tested because of close contact with patient A. She tested positive for COVID-19 on March 24; her nasopharyngeal swab sample C_t_ was ≈36.31 (≈10^3^ copies/mL) (Table). Two days afterward, she experienced diarrhea (Table).

**Figure 1:**
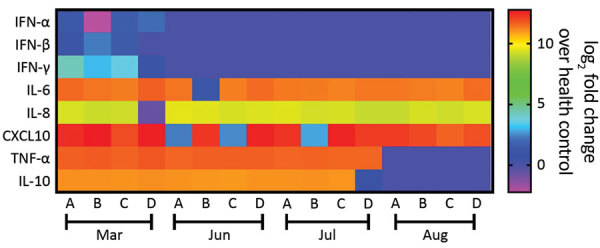
Heatmap showing the profile of innate immune response from patients who experienced 2 episodes of severe acute respiratory syndrome coronavirus 2 (SARS-CoV-2) infection, Brazil, 2020. We measured the mediators of innate immunity by ELISA for patients A–D. For comparison, these molecules were also quantified in the plasma from 5 healthy donors negative for SARS-CoV-2. The heatmap displays the log_2_ ratio of the fold-change from the plasma of the patients over the healthy volunteers. The means + standard error of the means for the healthy volunteers were the following: IFN-α = 20.4 + 4.7 pg/mL; IFN-β = 26.0 + 3.9 pg/mL; IFN-γ = 27.8 + 7.8 pg/mL; IL-6 = 13.4 + 1.7 pg/mL; IL-8 = 137 + 21.6 pg/mL; IL-10 = 165.4 + 40.7 pg/mL; TNF-α = 33.8 + 11.5 pg/mL; and CXCL-10 = 61.0 + 27.3 pg/mL. CXCL, C-X-C motif chemokine ligand IFN, interferon; IL, interleukin; TNF, tumor necrosis factor.

Patient B shares a household with patients C and D, a married couple, both 34 years old. Patients C and D were not in social isolation because of their work duties. Although patient C was asymptomatic, he displayed a C_t_ of 35.71 (10^3^ copies/mL) on March 25 (Table). Patient D was negative by molecular testing on March 26, but 1 week later, she had a detectable viral load (C_t_ 36.01, 10^3^ copies/mL) and reported diarrhea in the following days (Table). On March 27, all 4 patients experienced an increase of inflammatory mediators (interleukin [IL] 6, IL-8, and tumor necrosis factor α) and regulatory (IL-10) and chemotactic (C-X-C motif chemokine ligand 10) and antiviral (interferon γ) signals, relative to healthy SARS-CoV-2–negative controls (Figure 1). Although cytokine response was consistent with the resolution of the infection, the anti–SARS-CoV-2 neutralizing humoral response was not detected in late March 2020 (Table; Appendix Figure 2).

**Figure 2:**
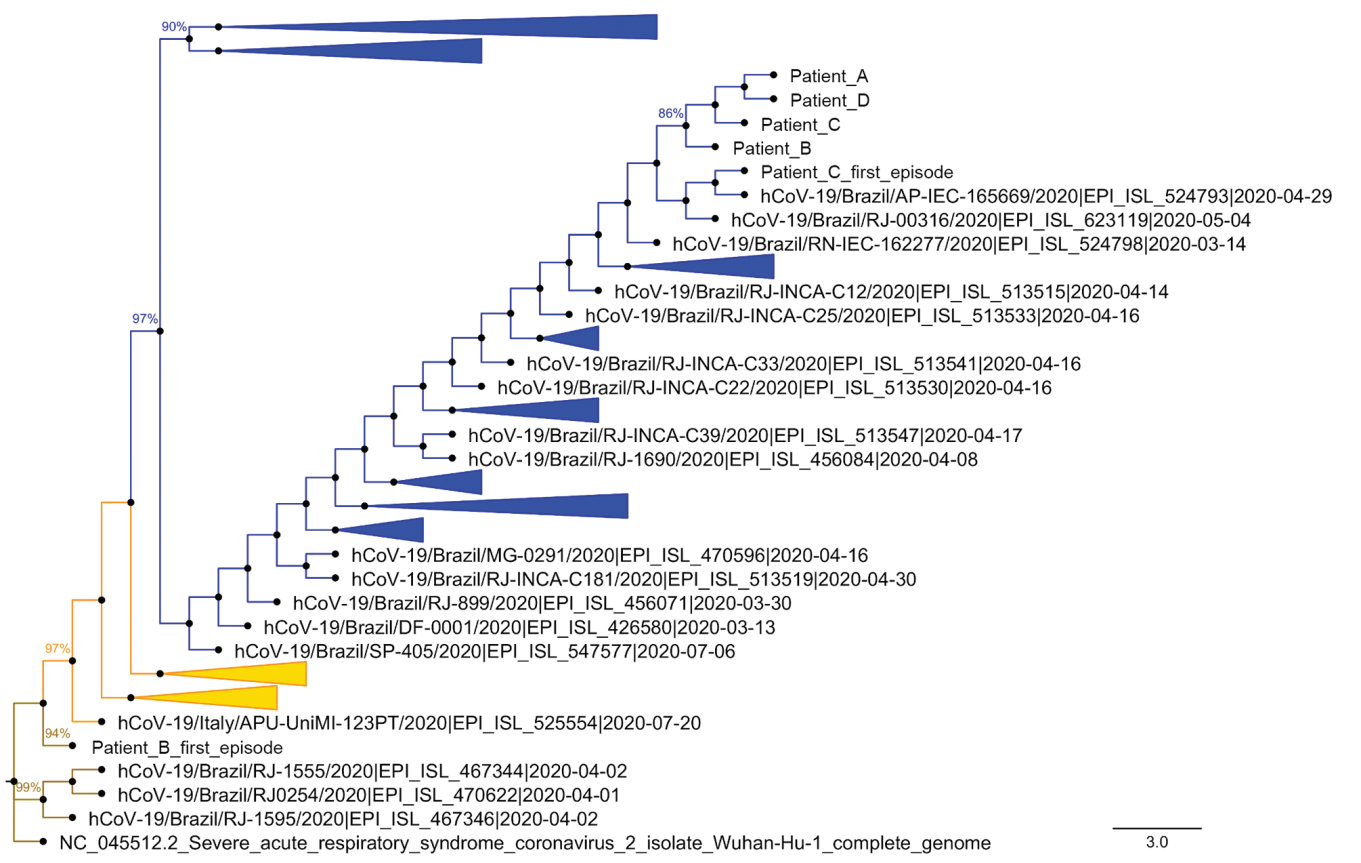
Phylogenetic analysis of severe acute respiratory syndrome coronavirus 2 genomes from reinfected patients, Brazil, 2020. Representative genomes deposited in GISAID (Appendix Table 1, Figure 3) were compared with sequences from virus genomes found in the respiratory samples from the first infection of patients B and C, and the second infection of patients A–D. A condensed phylogenetic tree rooted by reference genome Wuhan-Hu-1 (EPI_ISL_402125) was created with 1,000 bootstraps. Initial trees for the heuristic search were obtained automatically by applying neighbor-joining and BioNJ algorithms to a matrix of pairwise distances estimated using the Jukes-Cantor model (*24*), and then selecting the topology with a superior log-likelihood value. The tree with the highest log likelihood (−46487.36) is shown. The final dataset included a total of 29,920 positions. Evolutionary analyses were conducted in MEGA version 7.0 (*22**,**23*). Evolutionary history was inferred using the maximum-likelihood method and Jukes-Cantor model. Brown represents the emerging clade 19A, orange the clade 20A, and blue the clade 20B. Scale bar indicates substitutions per site. hCoV, human coronavirus.

For patients B and C, we were able to obtain a full-length SARS-CoV-2 genome (Table). Complete genome sequencing, with Phred quality score >30, composed of 140,000–20,000,000 reads and 100-fold to 10,000-fold coverage, argues against a false-positive RT-PCR result (Appendix Table 2, first column). For patients A and D, the samples were insufficient for sequencing. In March 2020, patients B was infected with emerging SARS-CoV-2 clade 19A and patient C with SARS-CoV-2 clade 20B, (Table; Figure 2; Appendix Figure 3). The detection of the 2 distinct lineages indicates that patients B and C were infected independently and did not transmit the virus to each other (Table; Figure 2; Appendix Figure 3). These distinct lineages were co-circulating in Brazil in March 2020 when multiple introductions of the SARS-CoV-2 occurred (*27*). Emerging clade 19A is associated with imported cases in Brazil, because of its proximity to the Wuhan-01 sequence (Figure 2; Appendix Figure 3). Indeed, detection of clade 19A in the sample from Patient B is consistent with household transmission from patient A, and his contact with the symptomatic traveler. Patient C, a police officer, was frequently exposed to various probable sources of contamination; he was infected with an emerging clade 20B virus, the most prevalent variant in Brazil, during December 2020 (Figure 2, Appendix Figure 3). All patients recovered from a mild COVID-19 episode and were retested in the first half of April, when they had negative RT-PCR results.

In the last week of May 2020, when COVID-19 cases in Rio de Janeiro were at the peak of the first wave of the pandemic (*28*), these 4 patients reported more signs and symptoms of SARS-CoV-2 infection than in March (Table). During the second episode, they experienced fever and cough, along with fatigue, headache, body ache, anosmia, and ageusia. Real-time RT-PCR revealed higher viral loads in the nasopharyngeal swab samples than at the time of the first infection: C_t_ of 21.76 (≈10^7^ copies/mL) for patient A, 21.84 (≈10^7^ copies/mL) for patient B, 26.38 (≈10^5^ copies/mL) for patient C, and 16.87 (≈10^9^ copies/mL) for patient D (Table).

On June 3, a week after the second episode, we detected SARS-CoV-2 immunoglobulins in patients A and B, but they had low to no neutralizing activity (Table; Appendix Figure 2). These serologic samples from June indicate that the first episode of COVID-19 was not followed by a sustained neutralizing humoral response, as judged by 90% PRNT (PRNT_90_) titers (Table). Because signals of a humoral effector memory were inconsistent after the first episode of COVID-19 (Table), we could speculate that the enhanced production of interferons and proinflammatory mediators led to resolution of the primo-infection (Figure 1). During the second episode of COVID-19, most of the cytokine levels were still higher than in healthy volunteers (Figure 1).

On July 9, forty days after the episode of reinfection, all patients had detectable immunoglobulin levels and their lowest PRNT_90_ results (Table; Appendix Figure 2), declining thereafter by August 10 (Table; Appendix Figure 2). In July, patients’ tests continuously showed upregulated pro-inflammatory markers (Figure 1), which are consistent with an enhanced response to a second SARS-CoV-2 exposure. In August, the markers of inflammation and regulatory responses, tumor necrosis factor α and IL-10, decreased compared with levels from previous months (Figure 1).

In the second episode, we fully sequenced the SARS-CoV-2 genome from all patients (Table; Figure 2; Appendix Table 2, Figure 3). SARS-CoV-2 sequences from the reinfection clustered together, suggesting a household transmission for patients A–D (Figure 2; Appendix Figure 3). The emerging genotype 20B, which was the main variant circulating in Brazil since May 2020, was detected in all samples from the second episode (Figure 2; Table; Appendix Figure 3). For patient B, the first episode was associated with the emerging clade 19A and the second with 20B (Figure 2; Appendix Figure 3). Two episodes provoked by genetically distinct lineages support the possibility of reinfection.

Although both episodes in patient C were associated with clade 20B, they clustered apart on the phylogeny with significant statistical support: by 86% of bootstrap using maximum likelihood (Figure 2) and by Bayesian inference (Appendix Figure 3). Genetic markers in the SARS-CoV-2 genome were different in the patient’s 2 episodes of COVID-19 (Appendix Table 2). The genomes diverge at the genes encoding the nonstructural protein (NSP) 3, 3C-like proteinase, and exonuclease (Appendix Table 2). In addition to the genetic variations, poor development of anti–SARS-CoV-2 serology between the 2 episodes of infection points suggests a reinfection scenario.

## Discussion

Seasonal human coronaviruses may cause reinfection, as documented for the past 35 years (*8*,*29*). Of note, in veterinary medicine, domestic mammals also have coronavirus reinfection (*30*). Adaptive, memory-generating immunity to coronaviruses is heterogeneously sustainable in mammals, and some events of infection are controlled at the level of the innate immunity (*31*–*33*). We fully documented reinfection in 2 genetically unrelated persons in Rio de Janeiro, Brazil, describing patients who sought care twice in a 2-month interval who received clinical and laboratory diagnosis of COVID-19. Virus polymorphisms from the primary and second episodes and negative RT-PCR between the events strengthen the argument toward reinfection. Neutralizing anti–SARS-CoV-2 titers were not detected during the first episode, nor at the baseline of the second episode, suggesting that patients were still vulnerable after the primary episode.

SARS-CoV-2 reinfection has been associated with new variants that overcome the immune response to natural infection, short-lasting humoral response, and a limited or absent neutralizing immunity after the primo-infection (*10*–*13*). The patients in Brazil described in this study are similar to cases in the United States and Ecuador (*10*,*13*), in which reinfection was associated with more symptoms. Antibody-dependent enhancement or exposure to higher amounts of the virus could be the reason for the change from asymptomatic or oligosymptomatic to syndromic. In our study, primary and second infections were caused by a strain carrying the D614G mutation in the spike protein, which has been associated with higher replication efficiency (*34*). We did not detect other contemporaneous changes in the spike protein, such as 69/70 deletion, K417N, E484K, N501Y, P681H, or the 17 unique mutations of the P1 variant, which precluded association with more virulent strains (*35*). Beyond the spike protein, we detected the V125F change in the NSP14 protein; V125F is a nonconservative mutation that might increase the volume in the loop between β-sheets number 5 and 6, which could affect its methyltransferase activity (*36*). The V125F mutation is unlikely to increase virulence in a second episode. On the other hand, changes in NSP6 protein (*37*) and open reading frame 6 mRNA (S. Sarif Hassan et al., unpub. data, https://doi.org/10.1101/2020.11.06.372227) might result in viral evasion from innate immunity.

The primary infections of patients B and C were associated with emerging clades 19A and 20B, indicating that the 2 cohabitants were infected independently. Indeed, while 1 patient was in social isolation, the others were working outside in the community. The cocirculation of these clades of SARS-CoV-2 is consistent with the COVID-19 databases in GISAID and the multiple introductions of the new coronavirus in Brazil (*27*). In the following months, emerging clade 20B was identified as the most prevalent genotype, representing 60% of the deposited genomes on GISAID. The detection of clade 20B on the second episode of COVID-19, by the end of May, is associated with the peak of the pandemic in Rio de Janeiro, Brazil (*28*).

Distinct clades of SARS-CoV-2 were found in the primary and secondary respiratory samples from patient B, supporting the notion of reinfection. For patient C, both the first and second detections of SARS-CoV-2 were associated with clade 20B. Although viral persistence could be imagined in this scenario, SARS-CoV-2 genomic sequences from the first and second episodes do not cluster together in the same branch, as they did for an immunocompromised patient that shed SARS-CoV-2 for 150 days (*38*). Thus, phylogeny does not support the interpretation of persistence, by different methods. By branching apart, SARS-CoV-2 genomes associated with patient C strengthen the chances of a relevant degree of variation (*39*), indicating the direction of reinfection. In the documented case of SARS-CoV-2 and human coronavirus NL63 reinfection, different episodes were genetically associated with similar viral clades or strains (*40*). Whereas the detection of 2 episodes of SARS-CoV-2 infection from patient C was separated by >60 days, prolonged virus shedding in the nasopharyngeal swab specimens from mild cases lasted for 22–46 days (*41*), which is further evidence against persistence.

Results of SARS-CoV-2 reinfection affirm that immune rechallenge may be necessary to achieve humoral protection and underscore that sustainability of the immune response may be heterogeneous. We documented that these patients with mild COVID-19 displayed an innate immune response composed of pro-inflammatory and regulatory signals. Although cytokine storm has been associated with severe COVID-19 (*42*), we interpret that in the case of our patients, the innate immune response might have led to infection resolution (*43*). Another possibility, not explored in detail here, is that cellular-mediated immunity could have contributed to the mild clinical outcome (*2*,*4*,*44*). The natural history of mild COVID-19 described for these patients might also be representative of many persons exposed to the first wave of the pandemic, leading to the hypothesis that they would also be susceptible to other episodes of SARS-CoV-2 infections, even without the challenge being imposed by new variants.

We determined, on the basis of 6 years of surveillance and follow-up of human coronavirus reinfections, that initial exposure was insufficient to elicit a protective immune response, imposing limited pressure on selection on new seasonal coronavirus variants (*40*). Similarly, our data on a small cluster of patients recapitulate this natural history of reinfection, which may also occur for SARS-CoV-2.

Appendix Additional information about study of patients reinfected with severe acute respiratory syndrome coronavirus 2, Brazil, 2020.
