# Supplementary material for: Genetic Evidence and Host Immune Response in Persons Reinfected with SARS-CoV-2, Brazil
Source: Emerg Infect Dis. 2021 May;27(5):1446–53. doi: 10.3201/eid2705.204912 (PMC8084520; doi:10.3201/eid2705.204912)
Supplement: Appendix — Additional information about study of patients reinfected with severe acute respiratory syndrome coronavirus 2, Brazil, 2020. [file 20-4912-Techapp-s1.pdf]

Article DOI: https://doi.org/10.3201/eid2705.204912

# Genetic Evidence and Host Immune Response in Persons Reinfected with SARS-CoV-2, Brazil

## Appendix

## Detailed Materials and Methods

### Measurement of Serum SARS-CoV-2 antibodies

For quantitative analysis of anti-SARS-CoV-2 spike protein IgM, IgA, and IgG antibodies, we performed the S-UFRJ test, as previously described (R.G.F. Alvim et al., unpub. data, https://doi.org/10.1101/2020.07.13.20152884). Briefly, high binding ELISA plates were coated with 50 µL of SARS-CoV-2 spike protein (4 µg/mL in phosphate-buffered saline [PBS]) and incubated overnight. The coating solution was removed, and 100 µL of PBS 1% BSA (blocking solution) was added, and the plate was incubated at room temperature (RT) for 1–2 h. The blocking solution was removed, and 50 µL of diluted 1:40 (PBS 1% BSA) patient serum specimens were added; subsequently, the specimens were serially 3-fold diluted in the plate, which was incubated at RT for 2 h. Then, the plate was washed with 150 µL of PBS (5×) and 50 µL of 1:10,000 goat anti-human IgG, IgA, and IgM (Fc)-horseradish peroxidase antibody (SouthernBiotech, https://www.southernbiotech.com) were added, and the plate was incubated for 1.5 h at RT. The plate was washed again with 150 µL of PBS (5×) and then treated with TMB (3,3‘, 5,5; -tetramethylbenzidine) (Scienco, http://www.scienco.bio.br) until the reaction was stopped with 50 µL of HCl 1N. The optical density (OD) was read at 450 nm with 655 nm background compensation in a microplate reader (Bio-Rad Laboratories, Inc., https://www.bio-rad.com).

### Quantification of Plasma Cytokine Levels

Plasma samples from the acute and convalescent phases of the 2 episodes of SARS-CoV-2 infection were collected in EDTA-containing tubes. Tubes were placed on ice and aliquoted. Commercial ELISA kits (R&D Systems, https://www.rndsystems.com) were used to measure interferon (IFN)-α, -β and -γ, interleukin (IL)-6, −8 and −10, tumor necrosis factor α (TNF-α) and

induced protein 10 (IP10)/C-X-C motif chemokine ligand 10 (CXCL-10). This panel of mediators provides evidence for host production of antiviral, pro-inflammatory, and regulatory responses.

#### Plaque Reduction Neutralization Test (PRNT)

To determine serum titers to block SARS-CoV-2 infection, miniaturized PRNT was performed. In brief, human serum was heat-inactivated (30 min, 56°C) before 2-fold serial dilutions (from 1:4 to 1:2,056). Diluted serum specimens were incubated with 100 PFUs (PFU) of SARS-CoV-2 (GenBank accession no. MT710714) for 1 h at 37°C in 96-well plates. Afterward, mixtures of serum and virus were incubated with Vero E6 cells ($2 \times 10^4$ cell/well) in 96-well plates for an additional 1 h at 37°C. Next, a fresh semisolid medium containing 2.4% of carboxymethylcellulose (CMC) was added to the wells, and cultures were maintained for 72 h at 37°C. Cells were fixed with 10% formalin for 2 h at room temperature and stained with crystal violet (0.4%). Endpoint dilution inhibiting 90% PFU ($PRNT_{90}$) was scored. As a control, to validate each assay, a back-titration of the mock-treated virus was included. Our quality control accepts the final readout of the virus input to be equivalent to 100 $\pm$ 20 PFU. Plaque numbers were scored in $\geq$3 independent experiments with technical replicates by 2 independent blinded readers to determine the $PRNT_{90}$ (Appendix Figure 1).

#### Molecular Diagnosis

The total viral RNA was extracted using QIAamp Viral RNA (QIAGEN, https://www.qiagen.com), according to the manufacturer's instructions. Quantitative RT-PCR was performed using GoTaq Probe qPCR and RT-qPCR Systems (Promega, https://www.promega.com) in a StepOne Real-Time PCR System (Thermo Fisher Scientific, https://www.thermofisher.com). Amplifications were carried out in 25 µL reaction mixtures containing 2× reaction mix buffer, 50 µM of each primer, 10 µM of the probe, and 5 µL of RNA template. Primers, probes, and cycling conditions used to detect the SARS-CoV-2 RNA were those recommended by the US Centers for Disease Control and Prevention (CDC) (*1*). The standard curve method was employed for virus quantification, using synthetic RNA for gene N (Microbiologics, Minnesota, USA). The $C_t$ values for this target were compared with those obtained with different cell amounts ($10^7$ to $10^2$), for reaction calibration.

#### Genomic Analysis

Total viral RNA from nasopharyngeal swabs was extracted using QIAamp Viral RNA (QIAGEN), with minor modifications (*2*). In brief, extraction was performed in 2 mL of sample/lysis buffer (1:1) without RNA carrier, and purified RNA was obtained after binding and

elution from a single silica column. For better yields, a 50-μL eluate was repetitively loaded (4×) to the same column. Tests were performed to evaluate if digestion with DNase I and depletion of rRNA enhanced the quantity/quality of SARS-CoV-2-related reads. Samples negative for SARS-CoV-2 and positive for Zika or chikungunya virus were included as controls.

To improve sequencing readout, an amplicon-based enrichment strategy was carried out with the ATOPlex SARS-CoV-2 Full-Length Genome Panel v1.0 (kindly donated by MGI Tech Co., https://en.mgi-tech.com). For library construction, RNA samples were first quantified with the Qubit RNA BR Assay Kit (Thermo Fisher Scientific), according to the manufacturer's instructions. Approximately 5 ng of each sample were then used as inputs to reverse transcription (RT) reactions, followed by two-step multiplex PCR-based genome amplification and dual adaptor indexing (i.e., barcoding) using proprietary primer sets. Products were purified with DNA Clean beads at a 5:6 volume ratio and subsequent washing steps with 80% ethanol. Next, individual libraries were quantified with the Qubit 1X dsDNA HS Assay Kit (Thermo Fisher Scientific) and homogeneously pooled to a total sum of 400 ng, before being submitted to denaturation, circularization, and digestion steps. Finally, single-stranded circular DNA library pools were converted to DNA nanoballs by rolling circle amplification and submitted to pair-end sequencing (100 nt) on the MGISEQ-2000 platform (recently named as DNBSEQ-G400; MGI Tech Co. Ltd).

Genomic sequences were quality-scored, filtered, trimmed, and assembled in contigs through a validated workflow for SARS-CoV-2 (*3*). Genomes were aligned with MAFFT (*4*) or ClustalW (*5*), and phylogenies were constructed with MEGA version 7.0 (*6*,*7*), using the Jukes-Cantor model for Maximum likelihood estimates, by applying neighbor-join and BioNJ algorithms (*8*). The tree with the highest log-likelihood was used. Alternatively, MrBayes version 3.2.7 (http://nbisweden.github.io/MrBayes) was used for Bayesian inference (*9*,*10*) with a relaxed clock model with a priori model testing using the gamma rates and invariant sites (G + I) nucleotide substitution model, selected by jModelTest version 1.6 http://darwin.uvigo.es/software/jmodeltest.htm (*11*,*12*). The tree was visualized and edited with FigTree version 1.4.2 (http://tree.bio.ed.ac.uk). SARS-CoV-2 clades were determined using the Web site https://clades.nextstrain.org. To categorize mutations and polymorphisms, the SARS-CoV-2 reference genome Wuhan-Hu-1 (GISAID EPI ISL no. 402125) was aligned to our sequences. The original sequences used in this work are publicly available on https://www.gisaid.org: GISAID EPI ISL nos. 636737, 636834–636838. The dataset included in the analysis contained representative sequences of the emerging clades associated with our

sequences, 19A and 20B, as well as sequences from the genome 20A as a negative control (Appendix Table 1).

**References**


1. Centers for Disease Control and Prevention. Research use only 2019-novel coronavirus (2019-nCoV) real-time RT-PCR primers and probes. 2020 [cited 2020 Nov 11]. https://www.cdc.gov/coronavirus/2019-ncov/lab/rt-pcr-panel-primer-probes.html

2. Metsky HC, Matranga CB, Wohl S, Schaffner SF, Freije CA, Winnicki SM, et al. Zika virus evolution and spread in the Americas. Nature. 2017;546:411–5. PubMed https://doi.org/10.1038/nature22402

3. Cleemput S, Dumon W, Fonseca V, Abdool Karim W, Giovanetti M, Alcantara LC, et al. Genome Detective coronavirus typing tool for rapid identification and characterization of novel coronavirus genomes. Bioinformatics. 2020;36:3552–5. PubMed https://doi.org/10.1093/bioinformatics/btaa145

4. Katoh K, Kuma K, Toh H, Miyata T. MAFFT version 5: improvement in accuracy of multiple sequence alignment. Nucleic Acids Res. 2005;33:511–8. PubMed https://doi.org/10.1093/nar/gki198

5. Larkin MA, Blackshields G, Brown NP, Chenna R, McGettigan PA, McWilliam H, et al. Clustal W and Clustal X version 2.0. Bioinformatics. 2007;23:2947–8. PubMed https://doi.org/10.1093/bioinformatics/btm404

6. Kumar S, Stecher G, Li M, Knyaz C, Tamura K. MEGA X: Molecular Evolutionary Genetics Analysis across computing platforms. Mol Biol Evol. 2018;35:1547–9. PubMed https://doi.org/10.1093/molbev/msy096

7. Felsenstein J. Confidence limits on phylogenies: an approach using the bootstrap. Evolution. 1985;39:783–91. PubMed https://doi.org/10.1111/j.1558-5646.1985.tb00420.x

8. Jukes TH, Cantor CR. Evolution of protein molecules. In: Mammalian protein metabolism. Vol. III. H. N. Munro, editor. New York: Academic Press; 1969. p. 21–132.

9. Huelsenbeck, J P, Ronquist F. MRBAYES: Bayesian inference of phylogeny. Bioinformatics. 2001;17:754–755

10. Ronquist, F, Huelsenbeck JP. MRBAYES 3: Bayesian phylogenetic inference under mixed models. Bioinformatics 2003;19:1572–1574.

11. Darriba D, Taboada GL, Doallo R, Posada D. jModelTest 2: more models, new heuristics and parallel computing. Nature Methods. 2012;9:772.

12. Guindon S, Gascuel O. A simple, fast and accurate method to estimate large phylogenies by maximum-likelihood. Systematic Biology. 2003;52:696–704

**Appendix Table 1.** Access codes for sequences used in tree construction compared with patients' virus sequences in study of patients reinfected with severe acute respiratory coronavirus 2, Brazil, 2020

| No. | Name | Accession code |
|---|---|---|
| 1 | hCoV-19/Wuhan/Hu-1/2019 | EPI_ISL_402125 |
| 2 | hCoV-19/Brazil/RJ-UFRJ-9331/2020\|EPI_ISL_492036\|2020–06–01 | EPI_ISL_492036 |
| 3 | hCoV-19/Brazil/RJ-INCA-C44/2020\|EPI_ISL_513551\|2020–04–17 | EPI_ISL_513551 |
| 4 | hCoV-19/Brazil/RJ-INCA-C39/2020\|EPI_ISL_513547\|2020–04–17 | EPI_ISL_513547 |
| 5 | hCoV-19/Brazil/RJ-INCA-C38/2020\|EPI_ISL_513546\|2020–04–17 | EPI_ISL_513546 |
| 6 | hCoV-19/Brazil/RJ-INCA-C37/2020\|EPI_ISL_513545\|2020–04–16 | EPI_ISL_513545 |
| 7 | hCoV-19/Brazil/RJ-INCA-C36/2020\|EPI_ISL_513544\|2020–04–16 | EPI_ISL_513544 |
| 8 | hCoV-19/Brazil/RJ-INCA-C34/2020\|EPI_ISL_513542\|2020–04–16 | EPI_ISL_513542 |
| 9 | hCoV-19/Brazil/RJ-INCA-C33/2020\|EPI_ISL_513541\|2020–04–16 | EPI_ISL_513541 |
| 10 | hCoV-19/Brazil/RJ-INCA-C31/2020\|EPI_ISL_513539\|2020–04–16 | EPI_ISL_513539 |
| 11 | hCoV-19/Brazil/RJ-INCA-C30/2020\|EPI_ISL_513538\|2020–04–16 | EPI_ISL_513538 |
| 12 | hCoV-19/Brazil/RJ-INCA-C29/2020\|EPI_ISL_513537\|2020–04–16 | EPI_ISL_513537 |
| 13 | hCoV-19/Brazil/RJ-INCA-C27/2020\|EPI_ISL_513535\|2020–04–16 | EPI_ISL_513535 |
| 14 | hCoV-19/Brazil/RJ-INCA-C25/2020\|EPI_ISL_513533\|2020–04–16 | EPI_ISL_513533 |
| 15 | hCoV-19/Brazil/RJ-INCA-C24/2020\|EPI_ISL_513532\|2020–04–16 | EPI_ISL_513532 |
| 16 | hCoV-19/Brazil/RJ-INCA-C23/2020\|EPI_ISL_513531\|2020–04–16 | EPI_ISL_513531 |
| 17 | hCoV-19/Brazil/RJ-INCA-C22/2020\|EPI_ISL_513530\|2020–04–16 | EPI_ISL_513530 |
| 18 | hCoV-19/Brazil/RJ-INCA-C14/2020\|EPI_ISL_513517\|2020–04–14 | EPI_ISL_513517 |
| 19 | hCoV-19/Brazil/RJ-INCA-C13/2020\|EPI_ISL_513516\|2020–04–14 | EPI_ISL_513516 |
| 20 | hCoV-19/Brazil/RJ-INCA-C12/2020\|EPI_ISL_513515\|2020–04–14 | EPI_ISL_513515 |
| 21 | hCoV-19/Brazil/RJ-INCA-C07/2020\|EPI_ISL_513514\|2020–04–08 | EPI_ISL_513514 |
| 22 | hCoV-19/Brazil/RJ-INCA-C06/2020\|EPI_ISL_513513\|2020–04–08 | EPI_ISL_513513 |
| 23 | hCoV-19/Brazil/RJ-DCVN1/2020\|EPI_ISL_509434\|2020–03–23 | EPI_ISL_509434 |
| 24 | hCoV-19/Brazil/RJ-899/2020\|EPI_ISL_456071\|2020–03–30 | EPI_ISL_456071 |
| 25 | hCoV-19/Brazil/RJ-2072/2020\|EPI_ISL_456104\|2020–04–16 | EPI_ISL_456104 |
| 26 | hCoV-19/Brazil/RJ-1921/2020\|EPI_ISL_456091\|2020–04–09 | EPI_ISL_456091 |
| 27 | hCoV-19/Brazil/RJ-1719/2020\|EPI_ISL_456088\|2020–04–06 | EPI_ISL_456088 |
| 28 | hCoV-19/Brazil/RJ-1702/2020\|EPI_ISL_456087\|2020–04–08 | EPI_ISL_456087 |
| 29 | hCoV-19/Brazil/RJ-1690/2020\|EPI_ISL_456084\|2020–04–08 | EPI_ISL_456084 |
| 30 | hCoV-19/Brazil/RJ-1627/2020\|EPI_ISL_456083\|2020–04–03 | EPI_ISL_456083 |
| 31 | hCoV-19/Brazil/RJ-1595/2020\|EPI_ISL_467346\|2020–04–02 | EPI_ISL_467346 |
| 32 | hCoV-19/Brazil/RJ-1555/2020\|EPI_ISL_467344\|2020–04–02 | EPI_ISL_467344 |
| 33 | hCoV-19/Brazil/GO-L19-CD410/2020\|EPI_ISL_476333\|2020–04–02 | EPI_ISL_476333 |
| 34 | hCoV-19/Brazil/PR-5620/2020\|EPI_ISL_541343\|2020–03–20 | EPI_ISL_541343 |
| 35 | hCoV-19/Brazil/SP-193/2020\|EPI_ISL_523984\|2020–04–19 | EPI_ISL_523984 |
| 36 | hCoV-19/Brazil/SP-240/2020\|EPI_ISL_524468\|2020–05–01 | EPI_ISL_524468 |
| 37 | hCoV-19/Brazil/PE-IAM1126/2020\|EPI_ISL_572379\|2020–05–07 | EPI_ISL_572379 |
| 38 | hCoV-19/Brazil/SC-L15-CD265/2020\|EPI_ISL_476259\|2020–03–29 | EPI_ISL_476259 |
| 39 | hCoV-19/Brazil/SP-L14-CD257/2020\|EPI_ISL_476253\|2020–03–27 | EPI_ISL_476253 |
| 40 | hCoV-19/Brazil/SC-0244/2020\|EPI_ISL_470653\|2020–03–18 | EPI_ISL_470653 |
| 41 | hCoV-19/Brazil/AL-837/2020\|EPI_ISL_427292\|2020–03–18 | EPI_ISL_427292 |
| 42 | hCoV-19/Brazil/SP-294/2020\|EPI_ISL_527862\|2020–03–30 | EPI_ISL_527862 |
| 43 | hCoV-19/Brazil/SP-L5-CAMPI91/2020\|EPI_ISL_476419\|2020–04–23 | EPI_ISL_476419 |
| 44 | hCoV-19/Brazil/SP-254/2020\|EPI_ISL_524469\|2020–05–05 | EPI_ISL_524469 |
| 45 | hCoV-19/Brazil/SP-436/2020\|EPI_ISL_603033\|2020–07–13 | EPI_ISL_603033 |
| 46 | hCoV-19/Brazil/SP-441/2020\|EPI_ISL_603038\|2020–07–20 | EPI_ISL_603038 |
| 47 | hCoV-19/Brazil/RS-0242/2020\|EPI_ISL_470651\|2020–03–20 | EPI_ISL_470651 |
| 48 | hCoV-19/Brazil/SP-356/2020\|EPI_ISL_547575\|2020–06–16 | EPI_ISL_547575 |
| 49 | hCoV-19/Brazil/SP-321/2020\|EPI_ISL_534316\|2020–05–02 | EPI_ISL_534316 |
| 50 | hCoV-19/South_Korea/KCDC2712/2020\|EPI_ISL_522491\|2020–07–11 | EPI_ISL_522491 |
| 51 | hCoV-19/Brazil/PR-5621/2020\|EPI_ISL_541344\|2020–03–19 | EPI_ISL_541344 |
| 52 | hCoV-19/Brazil/SP-437/2020\|EPI_ISL_603034\|2020–07–11 | EPI_ISL_603034 |
| 53 | hCoV-19/Brazil/SP-433/2020\|EPI_ISL_603030\|2020–07–11 | EPI_ISL_603030 |
| 54 | hCoV-19/Brazil/UN-HIAE-SP04/2020\|EPI_ISL_486429\|2020–03–20 | EPI_ISL_486429 |
| 55 | hCoV-19/Norway/3069/2020\|EPI_ISL_549084\|2020–08–10 | EPI_ISL_549084 |
| 56 | hCoV-19/Brazil/SP-370/2020\|EPI_ISL_583500\|2020–06–29 | EPI_ISL_583500 |
| 57 | hCoV-19/Brazil/RJ-UFRJ-9331/2020\|EPI_ISL_492036\|2020–06–01 | EPI_ISL_492036 |
| 58 | hCoV-19/Thailand/Bangkok-0071/2020\|EPI_ISL_445380\|2020–03–30 | EPI_ISL_445380 |
| 59 | hCoV-19/Brazil/SP-439/2020\|EPI_ISL_603036\|2020–07–10 | EPI_ISL_603036 |
| 60 | hCoV-19/Brazil/SP-440/2020\|EPI_ISL_603037\|2020–07–20 | EPI_ISL_603037 |
| 61 | hCoV-19/Brazil/SP-394/2020\|EPI_ISL_583503\|2020–06–22 | EPI_ISL_583503 |
| 62 | hCoV-19/Brazil/SP-398/2020\|EPI_ISL_603028\|2020–06–30 | EPI_ISL_603028 |
| 63 | hCoV-19/Brazil/SP-L5-CAMPI77/2020\|EPI_ISL_476416\|2020–04–23 | EPI_ISL_476416 |

| No. | Name | Accession code |
|---|---|---|
| 64 | hCoV-19/Brazil/SP-283/2020\|EPI_ISL_527860\|2020–04–17 | EPI_ISL_527860 |
| 65 | hCoV-19/Ecuador/USFQ-004/2020\|EPI_ISL_477014\|2020–03–30 | EPI_ISL_477014 |
| 66 | hCoV-19/Ecuador/USFQ-020/2020\|EPI_ISL_471267\|2020–04–17 | EPI_ISL_471267 |
| 67 | hCoV-19/Ecuador/USFQ-039/2020\|EPI_ISL_481245\|2020–04–17 | EPI_ISL_481245 |
| 68 | hCoV-19/Ecuador/USFQ-1112/2020\|EPI_ISL_486847\|2020–06–30 | EPI_ISL_486847 |
| 69 | hCoV-19/Ecuador/USFQ-110/2020\|EPI_ISL_486845\|2020–06–30 | EPI_ISL_486845 |
| 70 | hCoV-19/Italy/APU-UniMI-123PT/2020\|EPI_ISL_525554\|2020–07–20 | EPI_ISL_525554 |
| 71 | hCoV-19/Brazil/GO-L19-CD413/2020\|EPI_ISL_476336\|2020–04–02 | EPI_ISL_476336 |
| 72 | hCoV-19/Brazil/MG-0288/2020\|EPI_ISL_470593\|2020–04–09 | EPI_ISL_470593 |
| 73 | hCoV-19/Brazil/RJ-0251/2020\|EPI_ISL_470619\|2020–03–27 | EPI_ISL_470619 |
| 74 | hCoV-19/Brazil/GO-0209/2020\|EPI_ISL_470576\|2020–04–03 | EPI_ISL_470576 |
| 75 | hCoV-19/Argentina/PAIS-A0023/2020\|EPI_ISL_430814\|2020–04–17 | EPI_ISL_430814 |
| 76 | hCoV-19/Luxembourg/LNS9470500/2020\|EPI_ISL_434505\|2020–04–08 | EPI_ISL_434505 |
| 77 | hCoV-19/Mexico/HID-InDRE-54/2020\|EPI_ISL_576257\|2020–07–22 | EPI_ISL_576257 |
| 78 | hCoV-19/Mexico/CMX-INMEGEN-02/2020\|EPI_ISL_522873\|2020–07–31 | EPI_ISL_522873 |
| 79 | hCoV-19/Peru/LIM-UPCH-0146/2020\|EPI_ISL_568540\|2020–08–28 | EPI_ISL_568540 |
| 80 | hCoV-19/Peru/LIM-INS-138/2020\|EPI_ISL_536533\|2020–03–17 | EPI_ISL_536533 |
| 81 | hCoV-19/Peru/LIM-INS-078/2020\|EPI_ISL_536478\|2020–07–02 | EPI_ISL_536478 |
| 82 | hCoV-19/Peru/LIM-UPCH-0160/2020\|EPI_ISL_568553\|2020–08–28 | EPI_ISL_568553 |
| 83 | hCoV-19/CotedIvoire/BKE0891/2020\|EPI_ISL_614389\|2020–08–18 | EPI_ISL_614389 |
| 84 | hCoV-19/Peru/LIM-INS-120/2020\|EPI_ISL_536518\|2020–05–05 | EPI_ISL_536518 |
| 85 | hCoV-19/Peru/LIM-UPCH-0138/2020\|EPI_ISL_568534\|2020–08–27 | EPI_ISL_568534 |
| 86 | hCoV-19/Turkey/KOC-IST-OD5/2020\|EPI_ISL_613460\|2020–06–21 | EPI_ISL_613460 |
| 87 | hCoV-19/USA/WI-UW-1054/2020\|EPI_ISL_516480\|2020–07–30 | EPI_ISL_516480 |
| 88 | hCoV-19/USA/WI-UW-759/2020\|EPI_ISL_495464\|2020–07–06 | EPI_ISL_495464 |
| 89 | hCoV-19/Peru/LIM-UPCH-0147/2020\|EPI_ISL_568541\|2020–08–28 | EPI_ISL_568541 |
| 90 | hCoV-19/USA/MI-MDHHS-SC22181/2020\|EPI_ISL_614232\|2020–10–12 | EPI_ISL_614232 |
| 91 | hCoV-19/USA/VA-DCLS-1506/2020\|EPI_ISL_581508\|2020–07–28 | EPI_ISL_581508 |
| 92 | hCoV-19/Peru/LIM-INS-100/2020\|EPI_ISL_536499\|2020–07–04 | EPI_ISL_536499 |
| 93 | hCoV-19/Mexico/CMX-INMEGEN-03/2020\|EPI_ISL_522874\|2020–07–31 | EPI_ISL_522874 |
| 94 | hCoV-19/Peru/LIM-UPCH-0127/2020\|EPI_ISL_568523\|2020–08–28 | EPI_ISL_568523 |
| 95 | hCoV-19/Peru/LIM-UPCH-0129/2020\|EPI_ISL_568525\|2020–08–28 | EPI_ISL_568525 |
| 96 | hCoV-19/Peru/LIM-UPCH-0128/2020\|EPI_ISL_568524\|2020–08–28 | EPI_ISL_568524 |
| 97 | hCoV-19/Romania/Mioveni-24095/2020\|EPI_ISL_468156\|2020–05–08 | EPI_ISL_468156 |
| 98 | hCoV-19/Mexico/TLA-InDRE-57/2020\|EPI_ISL_576260\|2020–08–13 | EPI_ISL_576260 |
| 99 | hCoV-19/Mexico/ZAC-InDRE-72/2020\|EPI_ISL_576275\|2020–08–14 | EPI_ISL_576275 |
| 100 | hCoV-19/France/BRE-BR9068/2020\|EPI_ISL_613557\|2020–09–06 | EPI_ISL_613557 |
| 101 | hCoV-19/Singapore/1110/2020\|EPI_ISL_605819\|2020–10–25 | EPI_ISL_605819 |
| 102 | hCoV-19/Netherlands/ZH-EMC-552/2020\|EPI_ISL_577980\|2020–09–08 | EPI_ISL_577980 |
| 103 | hCoV-19/Netherlands/ZH-EMC-607/2020\|EPI_ISL_578035\|2020–09–17 | EPI_ISL_578035 |
| 104 | hCoV-19/Brazil/SP-405/2020\|EPI_ISL_547577\|2020–07–06 | EPI_ISL_547577 |
| 105 | hCoV-19/Brazil/DF-0001/2020\|EPI_ISL_426580\|2020–03–13 | EPI_ISL_426580 |
| 106 | hCoV-19/Brazil/SP-345/2020\|EPI_ISL_583495\|2020–06–13 | EPI_ISL_583495 |
| 107 | hCoV-19/Brazil/PI-0239/2020\|EPI_ISL_470613\|2020–03–19 | EPI_ISL_470613 |
| 108 | hCoV-19/Brazil/RN-IEC-162277/2020\|EPI_ISL_524798\|2020–03–14 | EPI_ISL_524798 |
| 109 | hCoV-19/Brazil/RJ-INCA-C34/2020\|EPI_ISL_513542\|2020–04–16 | EPI_ISL_513542 |
| 110 | hCoV-19/Brazil/DF-891/2020\|EPI_ISL_427298\|2020–03–22 | EPI_ISL_427298 |
| 111 | hCoV-19/Brazil/DF-862/2020\|EPI_ISL_427297\|2020–03–23 | EPI_ISL_427297 |
| 112 | hCoV-19/Brazil/SP-399/2020\|EPI_ISL_603029\|2020–06–22 | EPI_ISL_603029 |
| 113 | hCoV-19/Ireland/D-NVRL-72IRL12139/2020\|EPI_ISL_528465\|2020–08–12 | EPI_ISL_528465 |
| 114 | hCoV-19/Brazil/MG-0291/2020\|EPI_ISL_470596\|2020–04–16 | EPI_ISL_470596 |
| 115 | hCoV-19/Brazil/RJ-00318/2020\|EPI_ISL_623121\|2020–05–12 | EPI_ISL_623121 |
| 116 | hCoV-19/Brazil/BA-L17-CD359/2020\|EPI_ISL_476305\|2020–03–31 | EPI_ISL_476305 |
| 117 | hCoV-19/Brazil/RJ-INCA-C181/2020\|EPI_ISL_513519\|2020–04–30 | EPI_ISL_513519 |
| 118 | hCoV-19/Brazil/AP-IEC-165669/2020\|EPI_ISL_524793\|2020–04–29 | EPI_ISL_524793 |
| 119 | hCoV-19/Brazil/RJ-0263/2020\|EPI_ISL_470630\|2020–04–13 | EPI_ISL_470630 |
| 120 | hCoV-19/Brazil/RJ-00364/2020\|EPI_ISL_623167\|2020–05–04 | EPI_ISL_623167 |
| 121 | hCoV-19/Brazil/RJ-UFRJ-58271/2020\|EPI_ISL_492048\|2020–06–01 | EPI_ISL_492048 |
| 122 | hCoV-19/Brazil/DF-615i/2020\|EPI_ISL_427294\|2020–03–13 | EPI_ISL_427294 |
| 123 | hCoV-19/Spain/GA-IBV-98006079/2020\|EPI_ISL_541066\|2020–07–03 | EPI_ISL_541066 |
| 124 | hCoV-19/Brazil/RJ-00316/2020\|EPI_ISL_623119\|2020–05–04 | EPI_ISL_623119 |
| 125 | hCoV-19/Argentina/PAIS-A0024/2020\|EPI_ISL_430815\|2020–04–18 | EPI_ISL_430815 |
| 126 | hCoV-19/Argentina/Heritas_HG001/2020\|EPI_ISL_476496\|2020–04–22 | EPI_ISL_476496 |
| 127 | hCoV-19/Argentina/Heritas_HG006/2020\|EPI_ISL_476561\|2020–05–07 | EPI_ISL_476561 |
| 128 | hCoV-19/Argentina/Heritas_HG007/2020\|EPI_ISL_476565\|2020–05–09 | EPI_ISL_476565 |
| 129 | hCoV-19/Argentina/Heritas-HG023/2020\|EPI_ISL_615121\|2020–05–26 | EPI_ISL_615121 |
| 130 | hCoV-19/Brazil/RJ-1466/2020\|EPI_ISL_456081\|2020–04–06 | EPI_ISL_456081 |
| 131 | hCoV-19/Brazil/RJ0272/2020\|EPI_ISL_470638\|2020–04–17 | EPI_ISL_470638 |

| No. | Name | Accession code |
|---|---|---|
| 132 | hCoV-19/Brazil/RJ0256/2020\|EPI_ISL_470624\|2020–04–03 | EPI_ISL_470624 |
| 133 | hCoV-19/Brazil/RJ0254/2020\|EPI_ISL_470622\|2020–04–01 | EPI_ISL_470622 |
| 134 | hCoV-19/Brazil/RJ0251/2020\|EPI_ISL_470619\|2020–03–27 | EPI_ISL_470619 |
| 135 | hCoV-19/Brazil/RJ0248/2020\|EPI_ISL_470616\|2020–03–24 | EPI_ISL_470616 |
| 136 | hCoV-19/Brazil/RJ-0720/2020 | EPI_ISL_636836 |
| 137 | hCoV-19/Brazil/RJ-01020/2020 | EPI_ISL_636834 |
| 138 | hCoV-19/Brazil/RJ-06020/2020 | EPI_ISL_636737 |
| 139 | hCoV-19/Brazil/RJ-01020–2/2020 | EPI_ISL_636835 |
| 140 | hCoV-19/Brazil/RJ-0720.2/2020 | EPI_ISL_63683 |
| 141 | hCoV-19/Brazil/RJ-0920/2020 | EPI_ISL_63683 |

**Appendix Table 2.** Genetic characteristics of the SARS-CoV-2 sequences detected in patients who were infected twice, Brazil, 2020

| Characteristics of SARS-CoV-2 sequences | First episode of infection | | Second episode of infection | | | |
|---|---|---|---|---|---|---|
| | Patient B (% quasispecies) | Patient C (% quasispecies) | Patient A (% quasispecies) | Patient B (% quasispecies) | Patient C (% quasispecies) | Patient D (% quasispecies) |
| No. reads | >140K | >2.600K | >472K | >24.400K | >15.600K | >20.000K |
| Phred quality score | -Q35 | -Q36 | -Q33 | -Q36 | -Q36 | -Q36 |
| Depth of coverage, average ± SD | 103.5 ± 1.24 | 2018.85 ± 31.53 | 350.85 ± 4.04 | 18426.125 ± 279.91 | 11753.53 ± 257.32 | 10061.58 ± 3701.20 |
| Mutations | | | | | | |
| **NSP2 (AA)** | | | | | | |
| G1522A (none) | X | | | | | |
| **NSP3 (AA)** | | | | | | |
| A6866G (N1383D) | X | | | | | |
| C3037T (none) | X | X (24.7) | X | X | X | X |
| C6021T (P1101L) | X (25.44) | | | | | |
| 4151_4152delTT (L478X) | | X | | | | |
| C7164T (T1482I) | | X (25.1) | | | | |
| T7082A (S1455T) | | | X (25.3) | | | |
| A7384T (Q1555H) | | | X (25.3) | | | |
| **NSP4 (AA)** | | | | | | |
| C9569T (P339S) | | | X (25.3) | | | |
| **3C-like proteinase (AA)** | | | | | | |
| A10904G (S284G) | | X (25.1) | | | | |
| **NSP6 (AA)** | | | | | | |
| C11514T (T181I) | | | X | X | X | X |
| **RNA-dependent RNA polymerase (AA)** | | | | | | |
| G15406T (A656S) | X | | | | | |
| C14408T (P323L) | X (74.6) | X | X | X | X | X |
| A14836G (I466V) | X (24.77) | | | | | |
| **Helicase (AA)** | | | | | | |
| A17105G (H290R) | | | X (25.3) | | | |
| **3′-5′-Exonuclease** | | | | | | |
| G18412T (V125F) | | | X | X | X | X |
| G18180T (K47N) | | | | | | X |
| 18180_18181insTG (K47_D48insX) | | | | | | X |
| **EndoRNase** | | | | | | |
| A20265G (none) | X (25.44) | | | | | |
| **2’-O-Ribose methyltransferase** | | | | | | |
| A21415T (K53*) | X (24.76) | | | | | |
| **Surface glycoprotein** | | | | | | |
| A23403G (D614G) | X | X | X | X | X | X |
| T22619G (W353G) | | | X (25.3) | | | |
| **Membrane glycoprotein** | | | | | | |
| G26795T (M91I) | X | | | | | |
| G27112A (S197N) | X (25.01) | | | | | |
| A26555N (E11X) | X (25.44) | | | | | |
| G26556N /A26557N/ G26558N (E12X) | X (25.44) | | | | | |
| C26559N (L13X) | X (25.44) | | | | | |
| 27184_27228del TACAGTAAGTGACAA CAGATGTTTCATCTCGTTGACTTTCAGGTT (V221X) | | | | X (24.7) | | |

| Characteristics of SARS-CoV-2 sequences | First episode of infection | | Second episode of infection | | | |
|---|---|---|---|---|---|---|
| | Patient B (% quasispecies) | Patient C (% quasispecies) | Patient A (% quasispecies) | Patient B (% quasispecies) | Patient C (% quasispecies) | Patient D (% quasispecies) |
| **ORF6** | | | | | | |
| T27299C (133T) | | X | X | X (75.3) | X | X |
| 27184_27228delTACAGTAAGTGACAAC AGATGTTTCATCTCGTTGACTTTCAGGTT (M1_V9del) | | | | X (24.7) | | |
| 27196_27228delCAACAGATGTTTCATCTC GTTGACTTTCAGGTT (M1_V9del) | | | | | X (24.54) | |
| 27197_27226delAACAGATGTTTCATCTC GTTGACTTTCAGG (M1_Q8del) | | | | | X (25.56) | |
| 27197_27226delAACAGATGTTTCATCTC GTTGACTTTCAGG (V9X) | | | | | X (25.56) | |
| A27313G (K38E) | | | | | | X |
| **Nucleocapsid** | | | | | | |
| G28881A/G28882G (R203K) | | X | X | X | X | X |
| G28883C (G204R) | | X | X | X | X | X |
| T29148C (I292T) | | X | X | X | X | X |

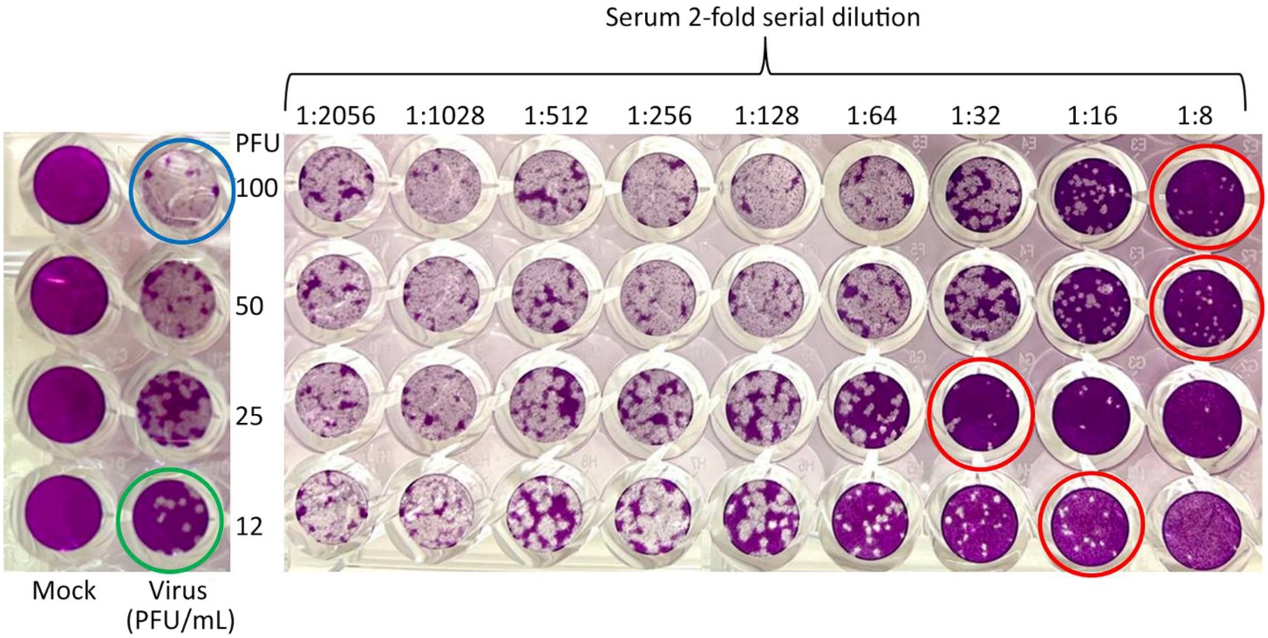


**Appendix Figure 1.** Representative readout of PRNT. 2-fold serial dilutions (from 1:4 to 1:2056) of human serum specimens were incubated in duplicates with ≈100 PFUs (PFU) of SARS-CoV-2. The serum–virus mixture was incubated for 1 h at 37°C and then added to Vero E6 cells ($2 \times 10^4$ cell/well) in 96-well plates and incubated for an additional 1 h at 37°C. Next, a medium with 2.4% CMC was added. After 72 h at 37°C, cells are fixed with 10% formalin and stained with crystal violet (0.4%). Mock = uninfected control. To calculate virus PFU/mL, a back-titration of the mock-treated virus was included in each experiment, the undiluted virus input incubated with the serum is highlighted by the blue circle, a 2-fold serial dilution of the virus is shown, the last dilution of the virus input (1:8) produced 12/13 PFU (green circle), validating the assay. The endpoint dilution of the serum capable of neutralizing the virus input (blue circle) by 90% was expected to produce ≈10 PFU; these dilutions are shown by the red circles.

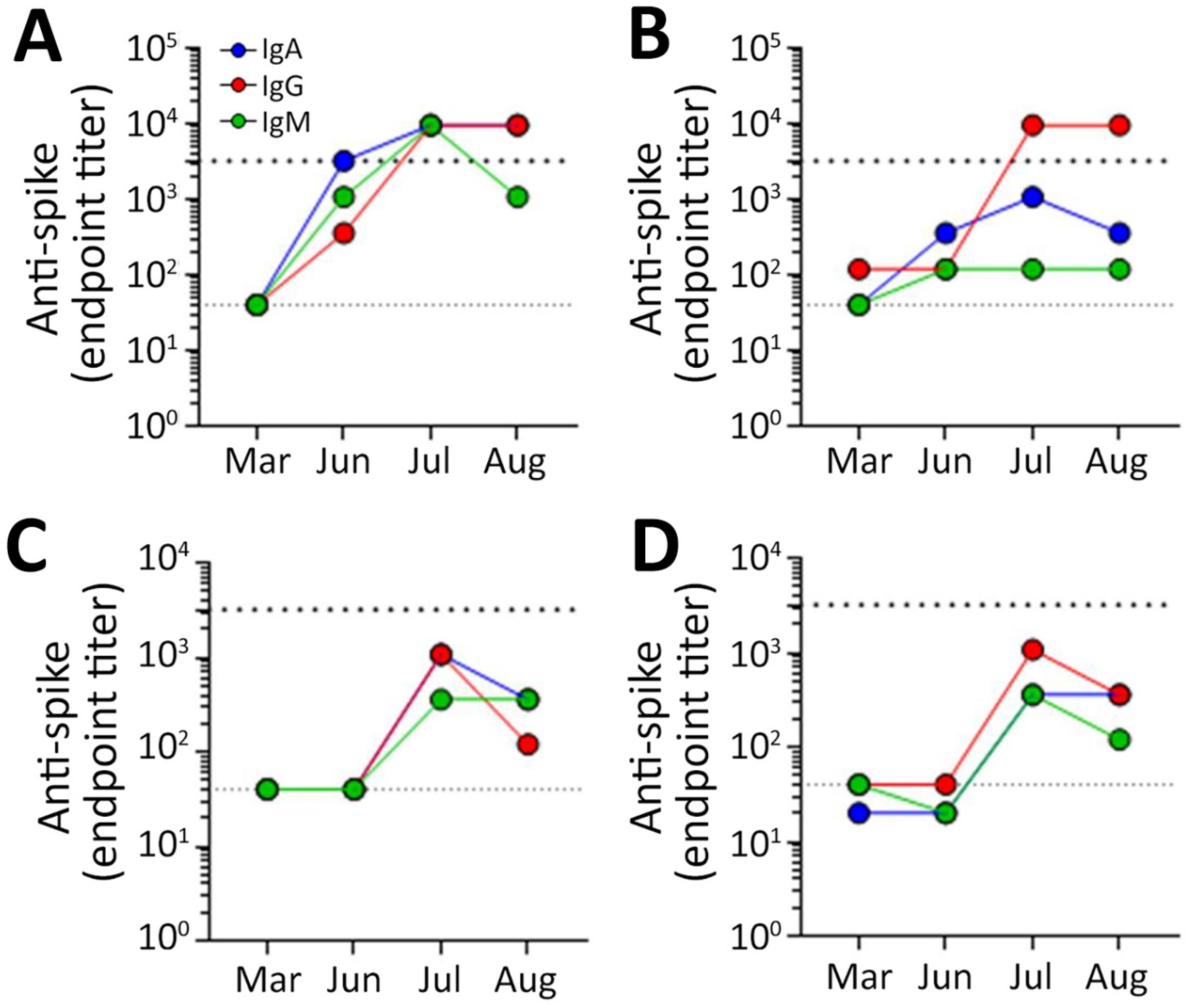


**Appendix Figure 2.** Quantitative analysis of IgA, IgM, and IgG from patients during the primary, second infections, and 2 months after the second infection. Plasma samples from patients were collected in March, June, July, and August for longitudinal detection of anti-Spike IgM (green), IgA (blue), and IgG (red) antibodies (A–D). The relative levels of antibodies were shown as endpoint titers of patient sample values for optical density [mean + 3 standard deviation (X + 3SD)] negative controls on the same ELISA plate. The dashed horizontal line represents the endpoint titer value between 10,000–30,000. The samples below the dotted line are considered negative.

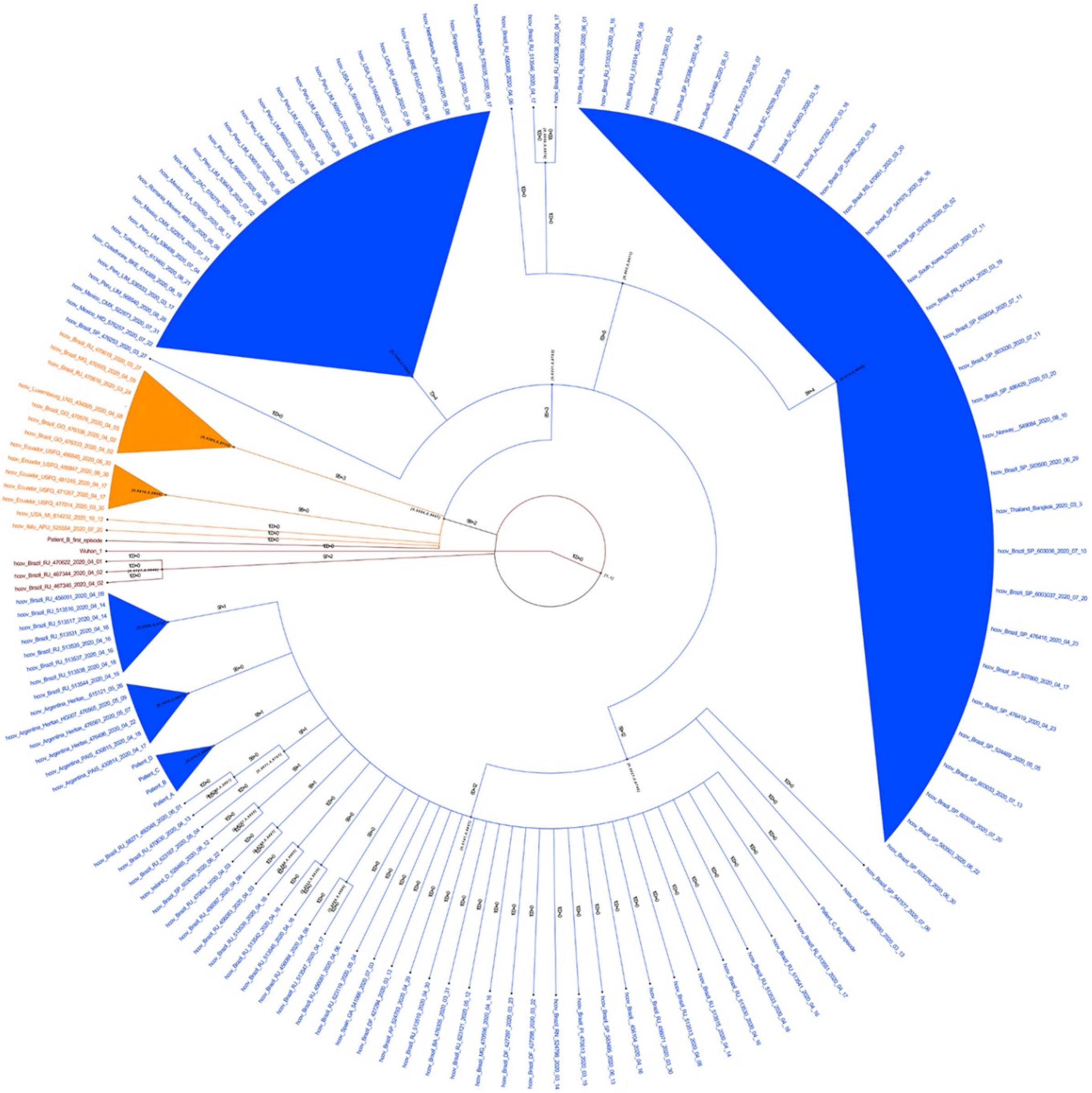

**Appendix Figure 3.** Phylogeny constructed by Bayesian inference using MrBayes version 3.2.7 (http://nbisweden.github.io/MrBayes), assuming a relaxed clock model with a priori model testing using the gamma rates and invariant sites nucleotide substitution model, selected by jModelTest version 1.6 (https://github.com/ddarriba/jmodeltest2). Emerging clade 19A is brown, clade 20A is orange, and clade 20B is blue. Posterior and anterior probabilities are presented for each branch.
